# Comparison of Phytochemical Constituents and Pharmacological Activities of Various Solvent Extracts Obtained from *Millettia speciosa* Stem Powder

**DOI:** 10.1155/2022/2486979

**Published:** 2022-11-16

**Authors:** Nasiruddin, Guangying Chen, Xiaobao Li, Ji Minghui, Tariq Masood, Waqas Safir, Muhammad Ali Khan, Muhammad Numan, Arsalan Khan, Muhammad Zeeshan, Shah Zeb

**Affiliations:** ^1^Key Laboratory of Tropical Medicinal Plant Chemistry of Hainan Province, College of Chemistry & Chemical Engineering, Hainan Normal University, Haikou 571127, China; ^2^Key Laboratory of Tropical Medicinal Resource Chemistry of Ministry of Education, Hainan Normal University, Haikou 571158, China; ^3^Agriculture Research Institute, Tarnab, Peshawar, Pakistan; ^4^Department of Agriculture Chemistry, The University of Agriculture, Peshawar, Pakistan; ^5^Department of Biochemistry & Molecular Biology, Xinjiang University, China; ^6^Department of Horticulture, Abdul Wali Khan University, Mardan, Pakistan

## Abstract

*Millettia speciosa* is a plant extensively used as an important component in Chinese herbal medicine and food-based medicines. The present study was carried out to determine the total flavonoid content (TFC), volatile phytoconstituents, and pharmacological activities, i.e., antityrosinase, sunscreen, and anticancer activity, of different fractions of *M. speciosa* stem. Different organic solvents of increasing polarity, i.e., petroleum ether (PE), ethyl acetate (EtOAc), and methanol (MeOH), were used for extraction. The highest total flavonoid content, i.e., 48.30 ± 0.90%, was reported for PE extract. Various important phytocomponents were revealed by gas chromatography-mass spectroscopy (GC-MS) analysis. Based on abundance, the major compounds were n-hexadecanoic acid (16.654%), n-hexadecanoic acid (14.808%), and beta-sitosterol (6.298%) for PE, EtOAc, and MeOH extract, respectively. The significant antityrosinase activity, i.e., 70.97 ± 0.66%, with an IC_50_ value of 4.58 mg/mL was noted for PE extract followed by EtOAc extract, i.e., 59.84 ± 0.67%, with IC_50_ value of 6.10 mg/mL. The maximum sunscreen activity was reported for PE extract exhibiting the maximum absorbance value (0.633 ± 0.06) in the ultraviolet (UV) region, i.e., UVC, while EtOAc extract showed the second highest level of absorbance in the UVB range, i.e., 0.632 ± 0.07. The strongest anticancer activity (49.73 ± 0.49% cell viability) towards MCF-7 breast cancer cell line was reported for PE extract with IC_50_ 197.51 *μ*g/mL. Our results confirmed the presence of potential therapeutic components for each extract with significant biological functions, showing the importance of the *M. speciosa* stem as a source of biomedicine. To our knowledge, this is the first report on *M. speciosa* stem extending comprehensive research about its phytochemical profile and various significant pharmacological activities.

## 1. Introduction

The use of plants as a source of food and shelter could be traced back to time immemorial, while their use as natural medicine is one of the oldest trends of healthcare known to humanity. The study of plants has made them a better choice to be investigated not only for food but also to find food-based medicines. A medicinal plant can be characterized to have potential therapeutic phytoconstituents that allow it to be used for curing or preventing a certain disease [1]. Numerous studies have explained the therapeutic potential of plants, which may include antitumor, anti-inflammatory, antidiabetic, antioxidant, and several other disorders [2]. The healing effects of herbal medicines are considered due to the presence of different bioactive compounds like alkaloids, polyphenols, flavonoids, terpenoids, and other important groups [3].

*Millettia speciosa* is a fabaceous plant used as an important ingredient in herbal and food-based medicines for its therapeutic functions. The use of *M. speciosa* as food can trace back to Ming (1368-1644) and Qing (1644-1912) Dynasties. According to famous Chinese traditional medicine monographs during Qing Dynasty, i.e., *Luchuan Bencao* and *Shengcao Yaoxing Beiyao*, it was used both as food and medicine. The development of herbal-based products for food and medicine has grown awareness about *M. speciosa* [4]. In China, *M. speciosa* is used to treat kidney weakness, frequent cough, and bronchitis. It is also consumed as traditional food and mixed with porridge and soup [5]. Cooking in soup may help release more important nutrients for bone and tendon strengthening. Previous chemical studies reported coumarins, alkaloids, flavonoids, terpenes, etc., making the main chemical composition of *M. speciosa* Champ. These components contribute significantly towards its therapeutic properties like hepatoprotection, antibronchitis, and immunity enhancement [6].

To date, no such study has been reported about the phytoconstituents and bioactivities of various extracts of *M. speciosa* stem powder and remained neglected. The aim is to gain new information on bioactive compounds in this part which could be used as a valuable material for new drug and functional food development. Therefore, this study sought to investigate the phytochemical composition and pharmacological activities of different extracts of *Millettia speciosa* stem for the first time. Second, folk healers have used *M. speciosa* to treat various human disorders but lacked scientific validation. Hence, the study was designed to confirm its folklore use and validate its therapeutic potential.

## 2. Materials and Methods

### 2.1. Collection of Plant Material

The stem part of *Millettia speciosa* was collected from Wanning city, Hainan province, in May 2018. The sample was thoroughly washed, shade dried, and ground into powder.

### 2.2. Extraction

Three distinct organic solvents, namely, PE, EtOAc, and MeOH, were used in succession to extract a 300 g powder sample. The sample received about 5 L of PE for 3 days and filtered through a cotton plug and finally through Whatman filter paper. For the following three days, the first step's leftover material was added back to 5 L of ethyl acetate. The EtOAc fraction was obtained using the same procedure. In order to obtain the crude extract of MeOH, the leftover residues were extracted using 5 L of methanol. The samples were vacuum-evaporated to dryness and kept at 4°C for further research [7].

### 2.3. GC-MS Analysis

GC-MS analysis of all three extracts was carried out by using an instrument model Agilent 7890A/5975C equipped with a capillary column HP-5 MS (30 m x 250 *μ*m x 0.25 *μ*m). The instrument was run in the Election Impact (EI) mode with an ionization voltage of 70 eV. Helium was employed as the carrier gas, flowing at a constant rate of 1.2 mL/min. Based on their retention times, the compounds were identified by comparing them with authentic standards and their mass spectral records found in the National Institute of Standards and Technology (NIST 08. L) Library [8].

### 2.4. Determination of Total Flavonoids

Total flavonoid content was determined by the colorimetric method [8]. The absorbance was measured at 510 nm by using a double-beam UV-Vis spectrophotometer (TU-1901, Beijing Puxi General Instrument Co., Ltd.), Different concentrations (0.008, 0.016, 0.024, 0.032, 0.040, and 0.048 mg/mL) of rutin were used to establish a standard calibration (*y* = 10.818*x* − 0.0217, *R*^2^ = 0.997). The extracts were dissolved with dimethyl sulfoxide, separately. In short, 0.5 mL of each extract was mixed with 2 mL of distilled water. Furthermore, 150 *μ*L of 5% NaNO_2_ solution was added. After five minutes, 600 *μ*L of 10% AlCl_3_ and 2 mL of 4% NaOH were added. The solution was thoroughly mixed, and distilled water was added to make the volume up to 5 mL. Total flavonoid content was calculated after 15 min as mg of rutin equivalent (RE) per gram dry weight of the extract. The formula is used as follows:

(1) $$\begin{array}{c} \text{Total flavonoid content}=\text{RE}\times \frac{V}{m}. \end{array}$$

### 2.5. Antityrosinase Activity

The assay was performed as reported by Park et al. [9] with slight alterations. Briefly, potato tyrosinase (1 mL) was mixed with 220 *μ*L of phosphate buffer (0.1 M, pH 6.5) and 2 mL of L-tyrosine and 2 mL of different concentrations of each extract. The reading was noted at 490 nm using a UV spectrophotometer after incubation for 30 min at 37°C. Arbutin and vitamin C were used as the positive control. Percent inhibition of tyrosinase was determined according to the following formula:

(2) $$\begin{array}{c} \%\text{Inhibition}=100-\left(\frac{{\text{Abs}}_{\text{control}}-{\text{Abs}}_{\text{sample}}}{{\text{Abs}}_{\text{control}}}\;\right)\times 100. \end{array}$$

### 2.6. Sunscreen Activity

The sun protective potential of all three samples was calculated by using the spectrophotometric method as described earlier [10]. Concentrations of 200 *μ*g/mL of the 03 test samples were prepared. The photoprotection activity was recorded in different regions of absorbance, i.e., UVC, UVB, and UVA, using a spectrophotometer (TU-1901, Beijing General Analysis Instruments). The positive controls (rutin and 4-methylbenzylidene camphor) were also run to demonstrate the validity of the results.

### 2.7. Anticancer Activity

#### 2.7.1. Cell Culture

The human breast cancer cell line (MCF-7) was received from Kunming Cell Bank, Chinese Academy of Sciences, China. The cells were cultured in Dulbecco's modified Eagle's medium (DMEM, Gibco, USA) having 10% fetal bovine serum (FBS, BOSTER, Wuhan, China) and 1% antibiotic penicillin (5000 units/mL, Biosharp, Hefei, China) which were then kept at 37°C in 5% CO_2_ humidified incubator. At 80% confluency, cells were subcultured, and a new medium was added every two to three days.

#### 2.7.2. Cellular Cytotoxicity Measurement

The assay described by Lamyae et al. was used with some modifications [11]. Cancer cells were plated in a 96-well plate at 4 × 10^3^ cells per well. After 24 hr incubation at 37°C, extracts of different concentrations were added and incubated for 72 hrs. After incubation, the medium was removed, and the crystal violet solution (100 *μ*L of 1%) was added along with fetal bovine serum (1 : 4.5) to each well for at least one and a half h. Each well received glacial acetic acid (30%) and gently mixed. The absorbance was measured using an automatic microplate reader (Thermo Scientific Multiskan Go) at 590 nm. The percent viability of cells was calculated with the following formula:

(3) $$\begin{array}{c} \%\text{Viability}=\frac{\text{absorbance of treated cells}}{\text{absorbance of control cells }}\times 100. \end{array}$$

Doxorubicin was used as the positive control (standard drug). The IC_50_ values were also calculated.

### 2.8. Statistical Analysis

The data obtained were analyzed using SPSS 22.0 software. A one-way analysis of variance (ANOVA) at *α* = 0.05 was carried out to establish the significance of the treatments, while IC_50_ values were calculated by GraphPad Prism™ 8.00.

## 3. Results

### 3.1. GC-MS Analysis

**Figure 1:**
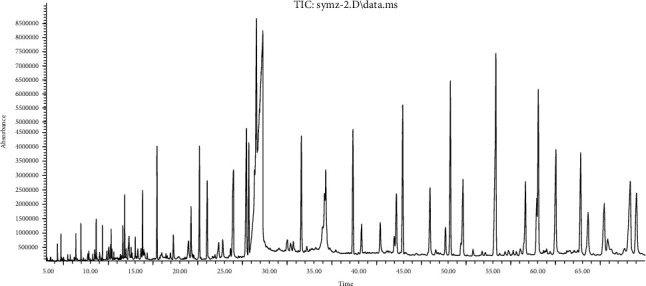
Total ion chromatogram for GC-MS analysis of PE extract of *M. speciosa* stem.

**Figure 2:**
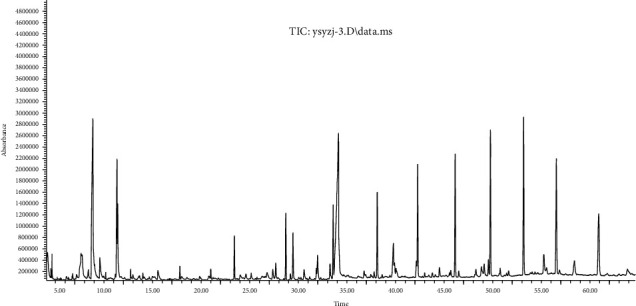
Total ion chromatogram for GC-MS analysis of EtOAc extract of *M. speciosa* stem.

**Figure 3:**
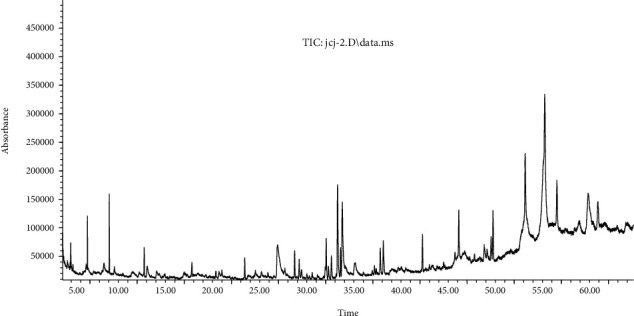
Total ion chromatogram for GC-MS analysis of MeOH extract of *M. speciosa* stem.

Investigation for bioactive compounds of *M. speciosa* stem was conducted according to experimental conditions as described in Section 2.3. GC-MS chromatograms for extracts of different solvents are given in Figures 1–3. The data for chemical compounds recorded at different retention times along with similarity index, molecular weight, and relative content are given in their respective Tables 1–3. The mass spectra of phytochemical constituents were compared with the National Institute of Standards and Technology (NIST) library to characterize and identify the number and nature of compounds.

The GC-MS chromatogram of petroleum ether extract depicted different peaks resulting from the presence of 57 compounds. Based on abundance, the top compound identified was n-hexadecanoic acid (16.654%), followed by hexadecanoic acid, ethyl ester (9.710%), octadecanoic acid (4.526%), cyclononasiloxane, octadecamethyl (3.209%), and octadecane (2.840%). The analysis of ethyl acetate extract resulted in 35 compounds based on their retention time. Out of these compounds, n-hexadecanoic acid (14.808%) was found a major chemical constituent followed by octadecanoic acid (2.288%), 2-pentadecanone, 6,10,14-trimethyl- (1.541%), and 7,9-di-tert-butyl-1-oxaspiro(4,5)deca-6,9-diene-2,8-dione (1.398%).

The GC-MS analysis of the methanolic extract of *M. speciosa* stem led to the identification of 13 chemical compounds. Among these compounds, beta-sitosterol (6.298%) was the most significant phytochemical. The other prevalent compounds were n-hexadecanoic acid (5. 369%), 4-((1E)-3-hydroxy-1-propenyl)-2-methoxyphenol (4.435%), and 1,2-benzenedicarboxylic acid, butyl 2-methylpropyl ester (3.566%). The compound n-hexadecanoic acid was recorded for all three extracts but was found as the most abundant for both PE and EtOAc extracts at different retention times. The overall composition of bioactive compounds of all three extracts was found significantly different. Those which were found similar have a significant variation in their amount of existence.

### 3.2. Total Flavonoid Content (TFC)

The total flavonoid content of various extracts of *M. speciosa* stem was measured by a spectrophotometric method which has been summarized in Table 4. The amount of flavonoid content in the tested extracts ranged from 6.30% to 48.30%. The results indicated the highest content (48.30 ± 0.90) for PE extract followed by EtOAc extract (28.30 ± 1.00).

### 3.3. Antityrosinase Activity

The tyrosinase inhibition properties of various extracts of *M. speciosa* stem were carried out, and IC_50_ values were calculated (Table 5). The results of all three extracts revealed an increase in tyrosinase inhibition values upon dose increment (Figure S1). The PE extract was the most effective and exhibited a maximum value of enzyme inhibition, i.e., 70.97 ± 0.66% with an IC_50_ value of 4.58 mg/mL. The second-highest antityrosinase activity was recorded for EtOAc extract which displayed an IC_50_ value of 6.10 mg/mL. The MeOH extract also showed inhibitory activity but the least.

### 3.4. Sunscreen Activity

The absorbance values of different extracts of *M. speciosa* stem recorded in three different ultraviolet (UV) regions, i.e., UVA, UVB, and UVC, are presented in Table 6 (Figure S2). The UV values indicated that all extracts have sunscreen capacity in all regions. The maximum value of absorbance (0.633 ± 0.06) was noted for PE extract in the UVC region. While in the UVB zone, the highest absorbance value, i.e., 0.632 ± 0.07, was noted for EtOAc extract. The overall order of the UV absorption of the tested extracts in all given zones was PE > EtOAc > MeOH. The results were not in comparison to that of pure compounds (rutin and 4-methylbenzylidene camphor); however, the EtOAc extract showed almost the same value (0.632 ± 0.07) in the UVB region compared to that of rutin, i.e., 0.663 ± 0.32.

### 3.5. Anticancer Activity

The cytotoxic activity of PE, EtOAc, and MeOH extracts against MCF-7 cells was carried out as presented in Table 7 (Figure S3). Doxorubicin was used as a positive control, and the response of the tested extracts was dose-dependent. Interestingly, no activity (100.00 ± 0.00% cell viability) was exhibited by all three extracts at the first two lower doses. The results revealed the highest cytotoxicity for PE extract, which killed almost 50% of the cancer cells by showing a cell viability value of 49.73 ± 0.49 percent with IC_50_ 197.51 *μ*g/mL. Meanwhile, EtOAc extract revealed 62.10 ± 1.11% cell viability means causing the death of about 38% of cancer cells with IC_50_ 263.17 *μ*g/mL, which is the highest anticancer activity after PE extract at the same dose level. The lowest effect was shown by MeOH extract, and the overall data ranged between 49.73 ± 0.49% and 100.00 ± 0.00% at the concentration from higher to lower, respectively.

## 4. Discussion

### 4.1. GC-MS Analysis

Studies about medicinal plants have shown their importance as a store for natural medicine. They are used both as a source of purified drugs and as such in folk medicine [12]. GC-MS is a proven and well-recognized technique to identify phytoconstituents along with other biologically important components like hydrocarbons, esters, and alcohols that exist in medicinal plants [13].

In the present study, GC-MS analysis of various extracts of *M. speciosa* stem was carried out. The most abundant compound in PE extract was n-hexadecanoic acid, which has anti-inflammatory, anticancer [14], antioxidant, and hypocholesterolemic properties [15]. Hexadecanoic acid ethyl ester has been reported to have antioxidant activities [16]. The compound octadecanoic acid was previously identified as an antibacterial, anticancer, and antiasthmatic agent [17]. Cyclononasiloxane, octadecamethyl- has also exhibited its bioactivity as an antifungal [18], while octadecane was detected as sesquiterpene hydrocarbon in the stem-bark extract of *Adansonia digitata* which has anti-inflammatory and antiallergic properties [19].

The EtOAc extract also demonstrated important biologically active compounds. The phytocomponent, 2-pentadecanone, 6,10,14-trimethyl- is one of the major compounds listed for EtOAc extract, which has demonstrated hypocholesterolemic, antioxidant, and lubrication properties [10]. The compound, 7,9-di-tert-butyl-1-oxaspiro(4, 5)deca-6,9-diene-2,8-dione was identified from EtOAc extract of *Penicillium citrinum* Strain ND7c and reported to have strong antimicrobial activities [20].

The GC-MS analysis also revealed various important bioactive compounds, but 4-((1E)-3-hydroxy-1-propenyl)-2-methoxyphenol and 1,2-benzenedicarboxylic acid, butyl 2 methylpropyl ester were found as major components. The former is a phenolic compound and is used for antifungal actions [21]. The latter, also called butyl isobutyl phthalate, has demonstrated antimicrobial and anticancer activities [22].

### 4.2. Total Flavonoid Content

Several studies have shown that flavonoids are responsible for various therapeutic activities like anticancer, hepatoprotective, antibacterial, and antidiabetic [23]. During the current study, the highest flavonoid content was reported for PE extract of *M. speciosa* stem, while the least amount was noted for MeOH extract, which was 8-fold less than that of PE extract. The difference in the quantity of flavonoid content depends on the polarity of the solvent and the flavonoids present in the plant extracts [24]. Several studies have been conducted to determine the flavonoid content of various extracts of whole and different plant parts. Mbinda and Musangi determined the flavonoid content of the methanolic extract of *Calotropis procera* stem and further established its antioxidizing and radical scavenging properties [25]. Furthermore, the study is supported by that of Ferdinand et al. who reported the flavonoid content of *Millettia laurentii* seed extract [26]. There are numerous studies about the flavonoid content of medicinal plants, which support our study and confirm the importance of the plant stem as a source of bioactive ingredients.

### 4.3. Antityrosinase Activity

Tyrosinase is a copper-containing enzyme with prime importance for controlling the production of melanin which is responsible for the hyperpigmentation of human skin. Therefore, the suppression of tyrosinase is an eminent approach to the development of melanogenesis inhibitors [27]. The current attempt was to find the tyrosinase inhibition potential of various *M. speciosa* stem extracts. The results indicated the antityrosinase activity of all extracts at different levels. The maximum inhibition was noted for PE extract followed by EtOAc extract. The results were supported by previous studies of *Tamarix nilotica* (Ehrenb.) Bunge stem extract, which demonstrated the L-tyrosine and L-DOPA inhibition values of 79.51% and 53.00%, respectively [28]. The stem extract of *Artocarpus chama* has shown strong antityrosinase activity both in enzymatic and intracellular assays [29]. Similarly, the stem extract of *Astragalus siliquosus* exhibited antityrosinase activity as reported by Zarei and his coworkers. However, the difference in inhibition level may be due to several factors like the composition of bioactive compounds, age of the plant, genetic and seasonal variations, and physiological and geographical factors [30].

### 4.4. Sunscreen Activity

Skin is a natural barrier between the internal parts of the body and the environment that protects against physical and chemical damage to skin tissues. Irreversible skin damage like skin cancer, aging, DNA damage, and oxidative stress can occur due to the presence of UV radiation and especially UVA and UVB. One of the protective measures to counter the effect of UV radiation is the use of medicinal plant extracts, which house natural antioxidants such as flavonoids and polyphenols. These compounds can absorb a wide range of UV light [31–33].

Our results were in good agreement with the available literature as all extracts showed sunscreen properties to some extent in all regions. The maximum photoprotection values were noted for EtOAc extract in the UVB zone while PE extract displayed the highest potential for UV absorption in the UVC region. The study of Preethima et al. confirmed the extract of *Pongamia pinnata* seeds to have high photoabsorbance properties in the UVA and B regions and hence can be used in sunscreen formulations [34]. The study could be further supported by Miguel et al. who reported higher photoabsorption capacity in the UVB range for the extracts of *Bejaria aestuans* and *Cavendishia pubescence* [35].

### 4.5. Anticancer Activity

Cancer is the second leading cause of death around the world. Comparing the side effects induced by synthetic drugs, plant-based natural products are a wise option [36]. During the current study, the anticancer activity of different extracts of *M. speciosa* stem powder against human breast cancer cells (MCF-7) was reported for the first time. The highest cytotoxicity value was recorded for PE extract followed by EtOAc at the same concentration of 200 *μ*g/mL. A similar study was carried out by Pham et al. which revealed the strong anticancer activity of crude extract of *Helicteres hirsuta* stem against MCF-7 cell lines [37]. Kumar and coscientists investigated *Millettia pinnata* for anticancer activity against lung cancer cells and found a higher cytotoxic effect of EtOAc extract [38]. The results obtained by Zingue et al. also declared notable anticancer activity of *Millettia macrophylla* extract against MCF-7 human breast cancer cells [39]. Several studies exist about the anticancer activity of plant extracts that correlate the anticancer activity with the combination of phytoconstituents present in a specific part of the plant.

The results clearly indicated that *M. speciosa* stem could be a potential source of natural anticancer products, which needs further studies related to isolation and clinical investigation to develop novel herbal-based anticancer medicine.

## 5. Conclusions

This was the first report about GC-MS analysis, antityrosinase, sunscreen, and anticancer activity of *Millettia speciosa* stem powder. Total flavonoid content was significantly different among all three extracts. The study revealed that various extracts of *Millettia speciosa* stem powder possess a very interesting phytochemical profile. More extractable metabolites were reported for petroleum ether extract. Ethyl acetate and methanolic extracts also showed considerable *in vitro* biological activities, but petroleum ether extract revealed the highest potential against tyrosinase, ultraviolet radiations, and cancer cell proliferation. The present study suggests that *Millettia speciosa* stem powder may be used as a potential source of natural products for pharmaceutical as well as nutraceutical development. However, future studies are needed to isolate bioactive compounds and *in vivo* studies to be carried out to establish a true cause-effect of *M. speciosa* stem.

## Figures and Tables

**Table 1:** GC-MS analysis of petroleum ether extract of *M. speciosa* stem.

No.	Rt (min)	Compound name	Similarity	MW	Rc (%)
1	3.594	Heptanal	97	114.104	0.023
2	4.727	Cyclotetrasiloxane, octamethyl-	91	296.075	0.146
3	5.477	3-Octen-2-one	95	126.104	0.068
4	5.755	2-Octenal, (*E*)-	93	126.104	0.043
5	6.388	Nonanal	94	142.136	0.224
6	6.957	Cyclopentasiloxane, decamethyl-	94	370.094	0.165
7	7.847	Decanal	90	156.151	0.065
8	9.049	Nonanoic acid	95	158.131	0.112
9	9.355	Cyclohexasiloxane, dodecamethyl-	93	444.113	0.172
10	10.327	2-Tetradecene, (*E*)-	97	196.219	0.268
11	10.432	Tetradecane	98	198.235	0.070
12	10.626	1,1-Dodecanediol, diacetate	91	286.214	0.067
13	11.495	Dimethyl phthalate	95	194.058	0.048
14	11.641	2,5-Cyclohexadiene-1,4-dione, 2,6-bis(1,1-dimethylethyl)-	98	220.146	0.244
15	11.842	Cycloheptasiloxane, tetradecamethyl-	91	518.132	0.420
16	11.995	Pentadecane	98	212.25	0.107
17	12.558	Phenol, 2,4-bis(1,1-dimethylethyl)-	96	206.167	0.215
18	13.308	1H-2-Benzopyran-1-one, 3,4-dihydro-8-hydroxy-3-methyl-	98	178.063	0.162
19	13.67	Dodecanoic acid	98	200.178	0.163
20	13.843	1-Hexadecene	98	224.25	0.562
21	13.989	Hexadecane	98	226.266	0.234
22	14.365	Hexadecanal	95	240.245	0.065
23	18.68	3,5-di-tert-Butyl-4-hydroxybenzaldehyde	92	234.162	0.034
24	18.972	Tetradecanoic acid	96	228.209	0.474
25	19.256	*E*-15-Heptadecenal	99	252.245	0.769
26	21.063	2-Pentadecanone, 6,10,14-trimethyl-	99	268.277	1.078
27	21.681	Oxacyclotetradecane-2,11-dione, 13-methyl-	91	240.173	0.063
28	22.349	Pentadecanoic acid	98	242.225	0.433
29	22.807	Benzenamine, *N*-[4-(1-methylethyl) benzylidene]-4-(1-pyrrolidylsulfonyl)-	90	356.156	0.455
30	23.981	7,9-Di-tert-butyl-1-oxaspiro(4,5)deca-6,9-diene-2,8-dione	99	276.173	2.136
31	24.572	Benzenepropanoic acid, 3,5-bis(1,1-dimethylethyl)-4-hydroxy-, methyl ester	91	292.204	0.028
32	25.441	1,2-Benzenedicarboxylic acid, butyl 2-methylpropyl ester	95	278.152	2.494
33	26.566	Hexadecanoic acid, ethyl ester	95	284.272	9.710
34	27.254	n-Hexadecanoic acid	99	256.24	16.654
35	30.381	Ethyl 14-methyl-hexadecanoate	91	298.287	0.384
36	34.307	Octadecanoic acid	95	284.272	4.526
37	38.31	Heptadecane	96	240.282	0.599
38	40.401	4,8,12,16-Tetramethylheptadecan-4-olide	98	324.303	0.862
39	41.27	1,2-Benzisothiazole, 3-(hexahydro-1H-azepin-1-yl)-, 1,1-dioxide	90	264.093	0.099
40	41.971	1-Docosene	99	308.344	0.456
41	42.208	Tetracosane	99	338.391	1.368
42	44.487	Decane, 3,6-dimethyl-	86	170.203	0.083
43	45.967	Pentacosane	99	352.407	1.563
44	46.606	1-Tricosene	95	322.36	0.163
45	47.1	Tetrapentacontane, 1,54-dibromo-	90	914.682	0.092
46	47.683	1,2-Benzenedicarboxylic acid, mono(2-ethylhexyl) ester	91	278.152	0.555
47	49.636	Hexacosane	99	366.423	1.909
48	56.626	Eicosane	95	282.329	1.821
49	57.877	(Z)-14-Tricosenyl formate	94	366.35	1.203
50	58.655	Octacosane	98	394.454	0.154
51	59.385	Pyridine-3-carboxamide, oxime, *N*-(2-trifluoromethylphenyl)-	92	281.078	0.151
52	60.017	Octadecane	96	254.297	2.840
53	61.268	2-Dodecen-1-yl(-)succinic anhydride	92	266.188	0.123
54	62.067	Heptacosane	93	380.438	0.290
55	63.617	Triacontane	98	422.485	1.388
56	65.423	Oxirane, hexadecyl-	93	268.277	1.832
57	68.335	Octadecane, 1-iodo-	97	380.194	3.166

**Table 2:** GC-MS analysis of ethyl acetate extract of *M. speciosa* stem.

No.	Rt (min)	Compound name	Similarity	MW	Rc (%)
1	5.186	Phenol	91	94.042	0.397
2	6.388	Phenol, 2-methoxy-	92	124.052	0.669
3	8.181	Decanal	91	156.151	0.155
4	10.745	Cyclohexasiloxane, dodecamethyl-	90	444.113	0.310
5	13.566	Vanillin	97	152.047	0.601
6	15.81	Cycloheptasiloxane, tetradecamethyl-	93	518.132	0.318
7	17.846	1H-2-Benzopyran-1-one, 3,4-dihydro-8-hydroxy-3-methyl-	97	178.063	0.253
8	18.805	Dodecanoic acid	94	200.178	0.230
9	18.993	1-Hexadecene	98	224.25	0.284
10	22.029	Benzaldehyde, 4-hydroxy-3,5-dimethoxy-	97	182.058	0.365
11	23.155	Octadecanal	92	268.277	0.297
12	24.6	2-Propenal, 3-(4-hydroxy-3-methoxyphenyl)-	90	178.063	0.173
13	24.781	4-((1*E*)-3-Hydroxy-1-propenyl)-2-methoxyphenol	99	180.079	0.666
14	25.379	Tetradecanoic acid	93	228.209	0.483
15	25.677	1-Octadecene	98	252.282	0.663
16	27.449	2-Pentadecanone, 6,10,14-trimethyl-	99	268.277	1.541
17	28.596	Pentadecanoic acid	99	242.225	0.549
18	29.979	7,9-Di-tert-butyl-1-oxaspiro(4,5)deca-6,9-diene-2,8-dione	99	276.173	1.398
19	31.264	Dibutyl phthalate	97	278.152	0.609
20	32.112	n-Hexadecanoic acid	99	256.24	14.808
21	34.773	Heptadecanoic acid	96	270.256	0.512
22	35.809	Phytol	90	296.308	0.234
23	36.983	Oleic acid	93	282.256	0.254
24	37.775	Octadecanoic acid	99	284.272	2.288
25	38.06	1-Docosene	96	308.344	0.598
26	41.798	Oxirane, heptadecyl-	93	282.292	0.236
27	42.521	4,8,12,16-Tetramethylheptadecan-4-olide	97	324.303	0.457
28	43.688	Octadecane	95	254.297	0.570
29	46.28	Heptadecane	96	240.282	0.457
30	47.128	Oxirane, hexadecyl-	94	268.277	0.680
31	47.559	1,2-Benzenedicarboxylic acid, mono(2-ethylhexyl) ester	91	278.152	0.601
32	52.061	13-Tetradecen-1-ol acetate	91	254.225	0.276
33	53.264	13-Docosenamide, (*Z*)-	97	337.334	1.370
34	53.541	Eicosane	96	282.329	0.588
35	56.404	1-Hexacosene	97	364.407	0.957

**Table 3:** GC-MS analysis of methanolic extract of *M. speciosa* stem.

No.	Rt (min)	Compound name	Similarity	MW	Rc (%)
1	4.727	Cyclotetrasiloxane, octamethyl-	91	296.075	1.126
2	7.041	Cyclopentasiloxane, decamethyl-	94	370.094	0.932
3	10.745	Cyclohexasiloxane, dodecamethyl-	95	444.113	0.608
4	11.072	2-Methoxy-4-vinylphenol	90	150.068	0.815
5	15.811	Cycloheptasiloxane, tetradecamethyl-	91	518.132	0.441
6	24.892	4-((*1E*)-3-Hydroxy-1-propenyl)-2-methoxyphenol	96	180.079	4.435
7	30.055	Hexadecanoic acid, methyl ester	98	270.256	1.724
8	30.604	Benzenepropanoic acid, 3,5-bis(1,1-dimethylethyl)-4-hydroxy-, methyl ester	90	292.204	0.769
9	31.264	1,2-Benzenedicarboxylic acid, butyl 2-methylpropyl ester	96	278.152	3.566
10	31.772	n-Hexadecanoic acid	99	256.24	5.369
11	35.788	Phytol	91	296.308	0.923
12	47.128	1-Eicosene	91	280.313	0.923
13	57.864	Beta-sitosterol	98	414.386	6.298

**Table 4:** Total flavonoid content of various *M. speciosa* stem extracts.

Extract	Total flavonoid content (%)
Petroleum ether	48.30 ± 0.90
Ethyl acetate	28.30 ± 1.00
Methanol	06.30 ± 0.90

**Table 5:** Antityrosinase activity and IC_50_ (mg/mL) of various *M. speciosa* stem extracts.

Compound	Concen. (mg/mL)	Petroleum ether		Ethyl acetate		Methanol	
		Inhibition (%) ± SD	IC_50_ (mg/mL)	Inhibition (%) ± SD	IC_50_ (mg/mL)	Inhibition (%) ± SD	IC_50_ (mg/mL)
Extract	0.5	31.22 ± 0.70	4.58	29.54 ± 0.73	6.10	13.72 ± 0.70	8.91
	2.0	36.29 ± 0.63		33.52 ± 0.65		19.49 ± 0.68	
	3.5	44.53 ± 0.62		38.79 ± 0.66		26.27 ± 0.63	
	5.0	51.67 ± 0.64		45.39 ± 0.55		32.57 ± 0.90	
	6.5	59.99 ± 0.72		52.61 ± 0.83		39.92 ± 0.72	
	8.0	70.97 ± 0.66		59.84 ± 0.67		46.67 ± 0.67	

Positive control	Concen. (mg/mL)						IC_50_ (mg/mL)

Arbutin	0.5	2.0	3.5	5.0	6.5	8.0	3.49
	Inhibition (%) ± SD						
	27.23 ± 0.29	38.19 ± 0.72	50.89 ± 0.56	62.16 ± 0.81	71.65 ± 0.66	82.65 ± 0.66	

Vitamin C	Concen. (mg/mL)						2.73
	0.5	2.0	3.5	5.0	6.5	8.0	
	Inhibition (%) ± SD						
	32.75 ± 0.59	44.35 ± 0.56	56.12 ± 0.52	67.69 ± 0.53	78.73 ± 0.59	92.89 ± 1.17	

**Table 6:** Sunscreen activity of various extracts of *M. speciosa* stem.

Compound	Concentration (*μ*g/mL)	UVA zone (320~400 nm)	UVB zone (280~320 nm)	UVC zone (200~280 nm)
Petroleum ether extract	200	0.291 ± 0.05	0.384 ± 0.05	0.633 ± 0.06
Ethyl acetate extract	200	0.369 ± 0.03	0.632 ± 0.07	0.459 ± 0.06
Methanol extract	200	0.212 ± 0.06	0.522 ± 0.09	0.355 ± 0.10
Rutin	40	1.014 ± 0.28	0.663 ± 0.32	1.908 ± 0.61
4-Methylbenzylidene camphor	40	0.512 ± 0.19	3.350 ± 0.52	3.485 ± 0.46

**(a):** 

Compound	Concen. (*μ*g/mL)	Petroleum ether		Ethyl acetate		Methanol	
		Viability (%) ± SD	IC_50_ (*μ*g/mL)	Viability (%) ± SD	IC_50_ (*μ*g/mL)	Viability (%) ± SD	IC_50_ (*μ*g/mL)
Extract	12.5	100.00 ± 0.00	197.51	100.00 ± 0.00	263.17	100.00 ± 0.00	404.82
	25	100.00 ± 0.00		100.00 ± 0.00		100.00 ± 0.00	
	50	88.20 ± 0.55		90.27 ± 0.58		93.94 ± 0.73	
	100	76.36 ± 0.51		85.42 ± 0.55		85.66 ± 0.68	
	200	49.73 ± 0.49		62.10 ± 1.11		77.28 ± 1.08	

**(b):** 

Positive control	Concen. (*μ*g/mL)					IC_50_ (*μ*g/mL)
Compound	1.25	2.50	5.00	10.00	20.00	10.95
	Viability (%) ± SD					
Doxorubicin	81.40 ± 0.54	73.24 ± 0.53	61.40 ± 0.52	50.34 ± 0.59	27.34 ± 0.61	

## Data Availability

All the data is available in the article. Figures have been submitted as supplementary materials to the journal.

## Supplementary Materials

Supplementary Materials Figure S1: antityrosinase activity of M. speciosa stem extracts, arbutin, and Vit C. Values are expressed as means ± SD (*n* = 3). Figure S2: sunscreen activity of M. speciosa stem extracts, rutin, and camphor. Values are expressed as means ± SD (*n* = 3). Figure S3: anticancer activity of M. speciosa stem extracts and doxorubicin. (A) MCF-7 cells were treated with different extracts of various concentrations. (B) MCF-7 cells were treated with standard drug, i.e., doxorubicin, as a positive control. Values are expressed as means ± SD (*n* = 3).
