# Supplementary material for: Comparison of Phytochemical Constituents and Pharmacological Activities of Various Solvent Extracts Obtained from Millettia speciosa Stem Powder
Source: Biomed Res Int. 2022 Nov 16;2022:2486979. doi: 10.1155/2022/2486979 (PMC9683946; doi:10.1155/2022/2486979)
Supplement: Supplementary Materials — Figure S1: antityrosinase activity of M. speciosa stem extracts, arbutin, and Vit C. Values are expressed as means ± SD (n = 3). Figure S2: sunscreen activity of M. speciosa stem extracts, rutin, and camphor. Values are expressed as means ± SD (n = 3). Figure S3: anticancer activity of M. speciosa stem extracts and doxorubicin. (A) MCF-7 cells were treated with different extracts of various concentrations. (B) MCF-7 cells were treated with standard drug, i.e., doxorubicin, as a positive control. Values are expressed as means ± SD (n = 3). [file 2486979.f1.docx]

FIGURE S1**:** Antityrosinase activity of *M. speciosa* stem extracts, Arbutin and Vit C. Values are expressed as means ± SD (n=3)

FIGURE S2: Sunscreen activity of *M.* *speciosa* stem extracts, rutin, and camphor. Values are expressed as means ± SD (n=3)

1. (B)

FIGURE S3: Anticancer activity of *M.* *speciosa* stem extracts and Doxorubicin. (A) MCF-7 cells were treated with different extracts of various concentrations. (B) MCF-7 cells were treated with standard drug i.e. Doxorubicin as a positive control. Values are expressed as means ± SD (n=3)
